# A Wearable, Multi-Frequency Device to Measure Muscle Activity Combining Simultaneous Electromyography and Electrical Impedance Myography

**DOI:** 10.3390/s22051941

**Published:** 2022-03-02

**Authors:** Chuong Ngo, Carlos Munoz, Markus Lueken, Alfred Hülkenberg, Cornelius Bollheimer, Andrey Briko, Alexander Kobelev, Sergey Shchukin, Steffen Leonhardt

**Affiliations:** 1 Medical Information Technology, RWTH Aachen University, Pauwelsstr. 20, 52074 Aachen, Germany; carlos.munoz@rwth-aachen.de (C.M.); lueken@hia.rwth-aachen.de (M.L.); hulkenberg@hia.rwth-aachen.de (A.H.); leonhardt@hia.rwth-aachen.de (S.L.); 2 Department of Geriatrics, University Hospital Aachen, 52074 Aachen, Germany; cbollheimer@ukaachen.de; 3 Department of Medical and Technical Information Technology, Bauman Moscow State Technical University, 105005 Moscow, Russia; briko@bmstu.ru (A.B.); ak.mail.ru@gmail.com (A.K.); schookin@bmstu.ru (S.S.)

**Keywords:** electrical impedance myography, bioimpedance, electromyogram, sensor fusion, muscle force, wearables, hardware design, multi-frequency

## Abstract

The detection of muscle contraction and the estimation of muscle force are essential tasks in robot-assisted rehabilitation systems. The most commonly used method to investigate muscle contraction is surface electromyography (EMG), which, however, shows considerable disadvantages in predicting the muscle force, since unpredictable factors may influence the detected force but not necessarily the EMG data. Electrical impedance myography (EIM) investigates the change in electrical impedance during muscle activities and is another promising technique to investigate muscle functions. This paper introduces the design, development, and evaluation of a device that performs EMG and EIM simultaneously for more robust measurement of muscle conditions subject to artifacts. The device is light, wearable, and wireless and has a modular design, in which the EMG, EIM, micro-controller, and communication modules are stacked and interconnected through connectors. As a result, the EIM module measures the bioimpedance between 20 and 200 Ω with an error of less than 5% at 140 SPS. The settling time during the calibration phase of this module is less than 1000 ms. The EMG module captures the spectrum of the EMG signal between 20–150 Hz at 1 kSPS with an SNR of 67 dB. The micro-controller and communication module builds an ARM-Cortex M3 micro-controller which reads and transfers the captured data every 1 ms over RF (868 Mhz) with a baud rate of 500 kbps to a receptor connected to a PC. Preliminary measurements on a volunteer during leg extension, walking, and sit-to-stand showed the potential of the system to investigate muscle function by combining simultaneous EMG and EIM.

## 1. Introduction

In the last decades, age-related diseases have become more frequent and are typically associated with movement impairments. The elderly/patients with movement impairments often face disability in achieving their daily living activities. Robotic devices have been developed to increase the efficiency of rehabilitation therapy and reduce costs. Current support systems are capable of providing more precise quantitative feedback, longer and more intensive training sessions, and improved functional outcomes compared to manual therapy [1,2]. In clinical practice, robotic devices and exoskeletons have emerged as mechanical rehabilitation tools attached to an able-bodied operator and augment the strength or endurance of movement for performing repetitive tasks [3]. To assess and control the interactive force between the robotic devices and the users in real-time, it is of importance to observe the user’s intention of motion and force [4]. The available options to estimate the interactive force-torque are limited [5]. One method is to apply the inverse model of the exoskeleton robot to derive the joint torques [6,7]. However, the parameters of the inverse models vary strongly among individuals, with time, and depending on the task, which makes robust impedance control challenging [8,9]. Alternative measurement technologies for estimation of the interactive human force/torque include sonomyography [10,11], optics [12], mechanomyography [13], magnetomyography [14], surface electromyography (EMG), and bioimpedance or electrical impedance myography (EIM).

Electromyography (EMG) is a common technique that measures the bio-potential produced by the contraction of muscles [15]. This technique detects the current generated by the ionic flow across the muscle fiber and transmitted through neighbor tissues. By using electrodes to connect to the body and proper instrumentation, an EMG quantifies the electrical activity of the muscle fibers in the vicinity of the electrodes during contraction. A well-known disadvantage of EMG is the projection of the measured EMG signal on a specific muscle, since surface EMG formation is a superimposition of all simultaneously stimulated muscles. An EMG signal does not necessarily reflect the total amount of force (or torque) that a muscle can generate since the number of motor units recorded by EMG electrodes will be less than the total number of motor units firing. Although the EMG “is one of the simplest electro-physiological signals to measure, it is also one of the most difficult to quantify” [16]. Currently, it is well accepted that the exclusive use of surface EMG is not sufficient to achieve a qualitative measurement of force/torque during muscle activities. In particular, measurements indicated that the EMG amplitude versus dynamic torque relationship for the superficial quadriceps femoris muscles does not demonstrate enough linearity and reliability to be used for examining the contributions of neural versus hypertrophic factors to training-induced strength gains [17].

Electrical impedance myography (EIM) is another non-invasive technique that measures bioimpedance (the di-electrical composition of tissues). The complex bioimpedance can be calculated by injecting harmless electrical alternating currents with a constant amplitude into the muscle through two electrodes and simultaneously measuring the resulting voltage through the other two electrodes (four-electrode configuration). Compared to EMG, EIM is potentially more resistant to artifacts. Different studies showed that EIM provides information not only on the parameters of the electrical activity of muscle fibers but also on the thickening and movement of the muscles itself [18,19,20]. The magnitude of the EIM signal and its change during muscle contraction/relaxation depends on the processes of changing the electrode-muscle position, as well as on the geometric parameters and electrical resistivity of the surrounding muscle tissue and the thickness and electrical resistivity of the skin-fat layer. EIM has also a high potential use in neuromuscular disorders [21,22] and in investigation of muscle fatigue [23,24]. While the use of EMG for bionic control of rehabilitation robotics has gained increasing interest in recent years [25,26], the use of EIM is still limited due to a lack of physiological interpretation of the EIM signal formation and lack of high-performance EIM hardware. A combination of simultaneous EIM and EMG can be helpful for a better understanding of EIM, since the muscle contraction component within the EIM signal can be detected and separated using EMG data. Recent studies have shown that the combination of EMG and bioimpedance may detect the muscle contraction more reliably [27,28,29] and may have a high potential for the detection and estimation of the muscle force/torque [30]. A wearable design that makes the device portable for physical activities such as walking, running, sit-to-stand, stairs climbing, etc. will be of great interest for a comprehensive investigation of muscle function.

Currently, there is a variety of bioimpedance and EMG devices with different sizes and performances on the market, for example, the Shimmer3 EMG Unit (Shimmer, Dublin, Irland), the Frequency Response Analyzer 1255B and 1294A (Ametek, Oak Ridge, TN 37831, USA), and the BIS SFB7 (ImpediMed Inc., Carlsbad, CA 92008, USA). However, there exist no commercial wearable devices which allow simultaneous measurement of EMG and EIM. Recently, we introduced the Integrated Posture and Activity Network by MedIT Aachen (IPANEMA), a wireless body sensor network (BSN) for healthcare applications and research. The current version of IPANEMA includes an inertial measurement unit, a force-sensing resistor on foot, and an environmental sensor [31,32]. This work introduces a novel hardware design for a wearable system that can acquire EMG and EIM signals simultaneously. Moreover, the operating frequency of the EIM can be set between 20–150 Hz during a measurement. Our novel design utilizes the AFE4300 and ADS1298 from Texas Instruments (Dallas, TX, USA) for the measurements of EMG and EIM. The device is light, wearable, wireless, and can be used as a new sensor node of IPANEMA BSN. This paper describes the requirements, hardware design, development, and validation process of the new device. For demonstrative purposes, we show preliminary measurements on a test subject during contraction of his upper legs on a test bench and performing sit-to-stand with and without some weights.

## 2. Materials and Methods

### 2.1. System Requirements

Our system is designed to investigate the activities of skeletal muscles of the upper and lower limbs via simultaneous EMG and EIM measurements. We aimed at using the standard four-electrode configuration for EIM measurement: two for current injection and two for voltage measurement. The EMG signal is captured simultaneously via the same measurement electrodes.

In most bioimpedance applications, such as body composition measurement, the typical frequency for the injecting current is 50 kHz, since it is more reactive in biological tissue [33]. However, EIM measurement at a wide range of frequencies would bring better insight into the nature of the EIM signal during muscle contraction of skeletal muscles, as later shown in our results. For the investigation of lower limb skeletal muscle, our system should perform EIM measurements over a larger frequency range between 2 and 200 kHz and utilize the current at different frequencies between 2 and 200 kHz. The system developed in this work will focus on deriving the magnitude of the bioimpedance signal $∥Z_{\mathrm{EIM}}\left[\Omega \right]∥$. The use of amplitude-modulated current at high frequency to investigate the human muscle contraction reduces the effect of motion artifacts, which are in the frequency range from 0.5 to 10 Hz [34].

The detected EMG signals have low amplitude, with a power spectrum mainly between 0 and 500 Hz [35]. The low amplitude of the EMG signal makes the technique sensitive when interfering with another measurement technique if applied simultaneously at the electrode-skin interface. It is also sensitive to motion artifacts, which affect the spectrum between 0 to 20 Hz and can be attenuated by a high-pass filter with a cut-off frequency between 10 to 20 Hz [36]. Additionally, the EMG signal should be low-pass filtered with a cut-off frequency at 150 Hz to remove high-frequency noise and capture the spectrum with the highest power density. Differential recording configurations amplify the difference between electrodes placed over the muscle of interest and take advantage of common-mode rejection, improving the signal’s quality against interference.

**Figure 1:**
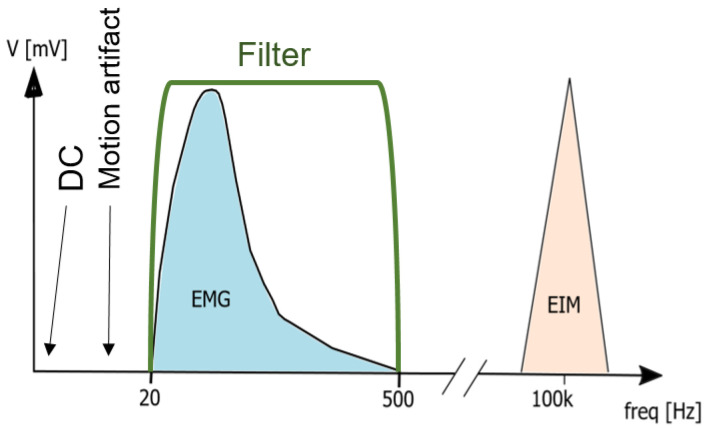
Spectrum of EMG and EIM signals captured at the same electrodes.

The EIM system developed in this work will focus on measuring the magnitude of EIM signal over a wide frequency range. While the most used frequency for the injecting current is 50 kHz, since it is more reactive in biological tissue [33], we use currents with frequencies between 2 and 200 kHz to assess the condition of the muscle. The use of amplitude-modulated current at high frequency to investigate human muscle contraction reduces the effect of motion artifacts, which are in the frequency range from 10 to 100 Hz [34] (see Figure 1). Consequently, the combination of EMG and bioimpedance detects the muscle contraction more reliably [27,29].

The EMG signal is vulnerable to noise and electromagnetic disturbance, which are coupled to the body through the skin and captured by the EMG module. For robustness against electromagnetic interference, the EMG module needs filters with a high common-mode rejection ratio or the use of a right leg drive (RLD). This technology senses the common-mode voltage at the amplifier’s output and feedbacks the common-mode voltage inverted in the patient’s body. The feedback current, and therefore the improvement in the CMRR, is limited by the amount of current injected into the patient’s body. This current must conform with the IEC60601-1.

Besides, the overall system has further requirements. According to the Nyquist criterion, the maximum frequency detected by the EMG module with a sampling frequency of 1000 Hz is 500 Hz. The aforementioned low-pass filter with a cut-off frequency of 500 Hz also acts as an anti-aliasing filter for the ADC built in the EMG module. An overall signal-noise ratio (SNR) of 30 dB or higher is also needed for performance. The EMG and EIM signal must have a delay between each other of less than 100 ms to allow the control of robotics [27]. The power supply is based on a buck converter that creates a split-rail voltage of ±5 V to supply the EMG module’s filters. The switching frequency of the buck converter must be high enough to reduce the influence of the EMI in the signals acquired by the system. The switching frequency for this application is 1.7 MHz. The EIM module performs the bioimpedance with an excitation signal between 2 and 200 kHz and an amplitude of less than 1 mA. The module must detect the bioimpedance between 20 and 200 Ω with a sampling frequency of 140 Hz. For control, the system needs a micro-controller with a clock frequency of 48 MHz or higher. The communication between the modules and the microprocessor uses the SPI protocol with a clock frequency of 4 Mhz or higher. The data is sent to a computer over serial or RF with a baud rate of 500 kbps. The settling time (the time needed to wait between the start of the injection of the excitation signal and the bioimpedance measurement) should be lower than 1000 ms. We note that a too-short settling time, however, increases the error sharply on the bioimpedance measurement. The final operation time, including measuring EMG and EIM, filtering, and sending the data over UART or RF should be less than 1 ms (system performance speed).

### 2.2. Modular Hardware Design

Our novel design uses the AFE4300 and ADS1298 of Texas Instruments to measure the EMG and EIM. Previous to the development of this work, no evidence was found of the combination of these chips to measure the EMG and EIM signals simultaneously without interference. Therefore, the system is separated into subsystems to simplify development and debugging (modular design). This design makes the system scalable and compact and allows the module’s affected hardware to be modified instead of changing the whole system.

**Figure 2:**
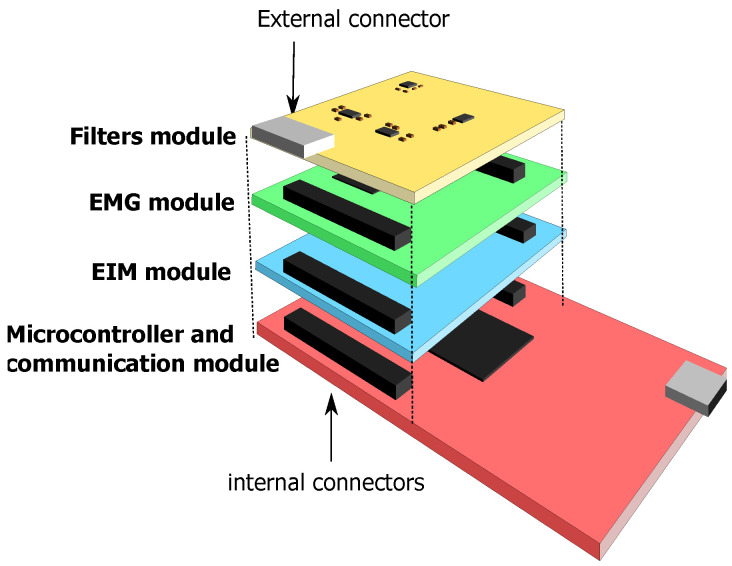
The modular hardware design.

The system’s main functionalities are integrated into separate modules that are stacked one on top of the other and connected through the internal connectors. Figure 2 depicts the modular hardware design of the system. The hardware is 7 × 5 × 5 cm (length × width × height) and can be enclosed in a small box attached with a belt to the person under test.

**Figure 3:**
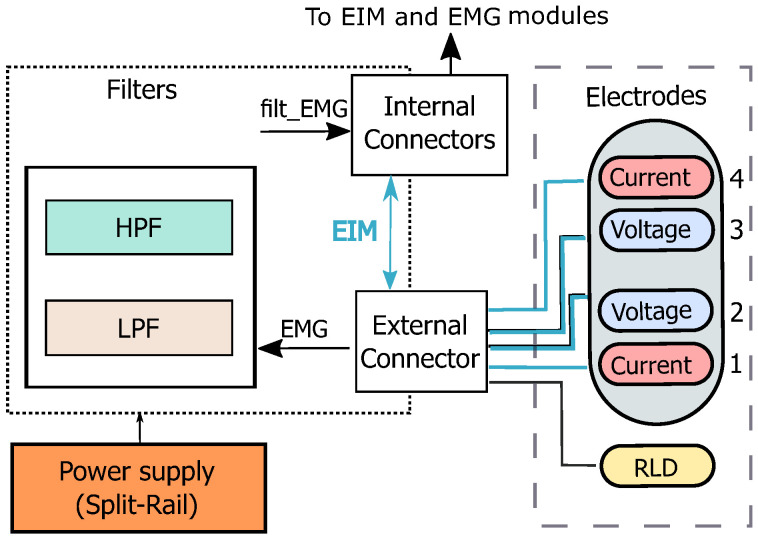
Block diagram of electrodes and filter modules.

The design includes three main modules: EMG, EIM, and filters. Figure 3 depicts the block diagram of the system, including electrode configuration. The device utilizes the common tetra-polar measurement principle for bioimpedance. Electrical currents are injected through the outer current, and electrical voltages are measured through the inner electrodes. The EMG and EIM voltage signals are captured by the same electrodes. The position of the electrodes attaching to the subject’s body can be chosen flexibly regarding the underlying investigation of muscle function. A RLD circuit is added to reduce common-mode interference.

**Figure 4:**
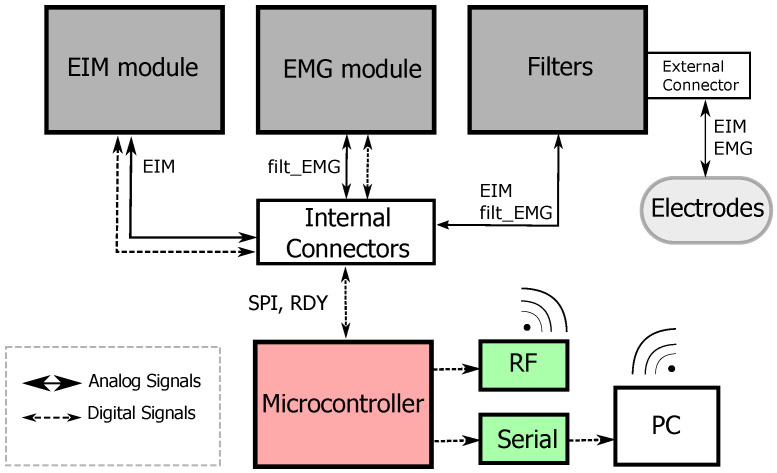
Overall structure of the EMG-EIM system.

For separation of the measured EMG and EIM signals, the system includes a filter module with fully-differential input filters. This module also delimits the spectrum of the EMG signal and prepares it to be acquired by an ADC. The EIM signals, on the other hand, pass through this module unfiltered. Once the signals have been separated, the EMG and EIM signals are then captured and digitized by the EMG and EIM modules. Figure 4 depict the overall structure of the modules. The micro-controller and communication module gets the data from the EMG and EIM modules over SPI. This module also can send the captured information in a data package over RF to a receiver connected to a PC for further signal processing.

#### 2.2.1. EMG and Filters Modules

The measurement of the EMG requires two modules: the filter module that builds the input high-pass and lowpass filters, and the proper EMG. Both modules are connected through the internal connector with the micro-controller. The core of the EMG module is based on the chip ADS1298 (Texas Instruments, Dallas, TX, USA). This module gets the filtered EMG analog signals from the internal connectors. The analog signals are digitized by the sigma-delta ADC built in the chip with a sample rate of 1 kSPS.

**Figure 5:**
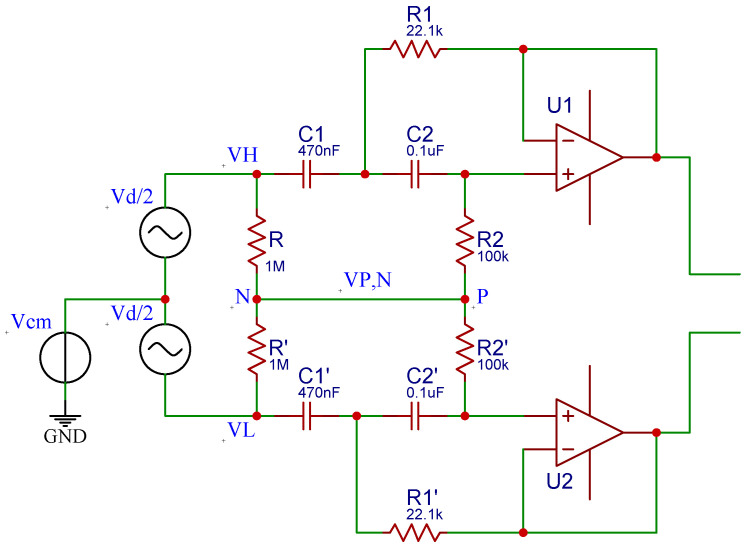
Schematic of the active high-pass filter, adapted from [37].

**Figure 6:**
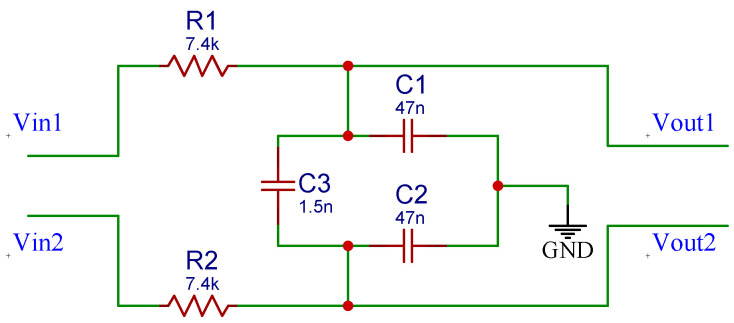
Schematic of the low-pass filter.

The high-pass filter used for this system is based on the fully differential active filter with a Sallen—Key topology proposed by Massarotto et al. [37]. This filter, depicted in Figure 5, was modified to meet the requirement for the lower cut-off frequency ($f_{c}=20$ Hz). The resistors and capacitors used for this filter have tolerances of 1% and 5%, respectively. Moreover, the filters are powered with a split-rail converter TPS65133 (Texas Instruments, Dallas, TX, USA). This chip is based on a boost converter that creates a split-rail voltage of ±5 V. The low-pass filter for the EMG signal is a simple RC full-differential passive filter of order 1 and is depicted in Figure 6.

#### 2.2.2. EIM Module

The EIM module makes use of the chip AFE4300 (Texas Instruments, Dallas, TX, USA) to perform the four-electrode bioimpedance measurement: two electrodes to inject the excitation current into the patient’s muscle and two electrodes to measure the excitation’s signal that travels through the muscle. Since only the magnitude of the bioimpedance is required for our application, the AFE4300 works in AC rectification mode. The module can measure bioimpedance between 20 and 200 Ω.

#### 2.2.3. Micro-Controller and Communication Modules

The module that controls the system’s functions is based on the development board SimpleLink Sub-1 GHz CC1310 (Texas Instruments, Dallas, TX, USA). This board has a 32-bit ARM Cortex-M3 processor that runs at 48 MHz. The processor includes several peripherals such as analog and digital inputs, serial (UART), and SPI ports. The board also builds an antenna that works at 868 MHz (ISM band allowed in Europe). The microprocessor collects data from the EMG and EIM modules and sends them over RF or serial at a baud rate of 500 kbps to a computer.

### 2.3. Software

**Figure 7:**
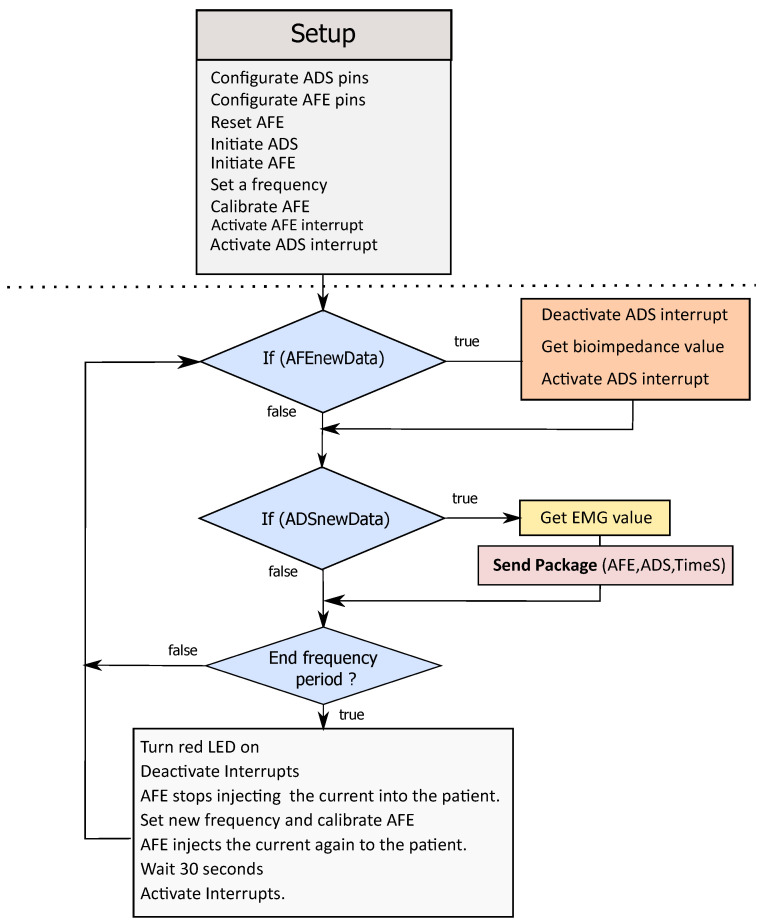
Main program structure.

The micro-controller CC1310 (Texas Instruments, Dallas, TX, USA, programmed with Energia) controls the system’s processes. To control the EMG and EIM module and reduce the program’s complexity, we wrote two libraries (EMG and EIM libraries) for the ADS1298 and AFE4300. After setting the register pins in the initialization step, the EIM module has to be calibrated using two calibration resistances, 200 Ω and 20 Ω, both produced by the company Vishay (Malvern, Pennsylvania, USA) with 1% tolerance. The raw value read by the AFE4300 at the calibration resistors is used to calculate the slope and offset of the linear calibration function. Figure 7 depicts the main program structure of the software.

The data can be transferred to the receiver over UART or RF. While transfering the data over UART, the values of the EIM (float type), EMG (float type), and time-stamp (long type) are converted from float and long to a character string. During package building, each value is separated by a comma character. The package is ended with an LF (new line feed) and CR (carrier return) character. Transferring the data in char increases the number of bytes to be transferred. However, some PC programs that capture serial data streams and plot the data in real-time, like SerialPlot or Arduino serial Monitor, require char data streams. The baud rate for the UART communication is set to 500 kbps. This baud rate reduces the time needed to transfer the data and achieve the performance required for this system. The serial data stream is captured on the PC side by a program written in Processing that saves the data in a text file. This data is then processed in MATLAB (Mathworks, MA, USA).

**Figure 8:**
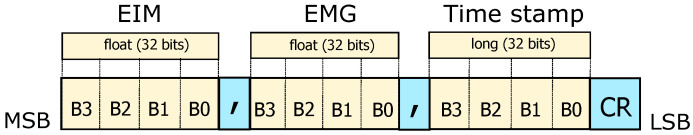
Package structure for RF transmission.

The data package structure in the RF transmission consists of the EIM, EMG, and time-stamp values separated by a comma character and CR character that ends the package. The values are split into four bytes. On the receiver side, the byte stream must be reconstructed. The receiver reads four bytes and combines them until it finds a comma character. The EIM and EMG result is reconstructed as a float type, and the time-stamp is reconstructed as a long type. This process is called serialization. Figure 8 shows the package structure for RF transmission.

### 2.4. Test Design

#### 2.4.1. System Performance Tests

The following tests were set up and evaluated:

- Measurement of CMRR using four precision resistors;
- SNR test with a function generator (Tektronik AFG 3022B) and sine waves at the frequencies 20, 50, 100, 150, 200, 300, 400, and 500 Hz;
- Frequency response analysis test. To evaluate the frequency responses of the system, we used a function generator to apply several frequencies between 20 and 500 Hz into the filters and an oscilloscope (Rigol DS1052e) to measure the filter’s output;
- Resistance measurement accuracy compared with the commercial bioimpedance device SFB7 (ImpediMed, Brisbane, Australia). We connected the device with known resistance (20, 32, 51, 68, 82, 109, 120, 129, 149, 170, 200 Ω with 5% tolerance) and applied tetra-polar measurements at 5, 10, 20, 30, 50, 100, 150, and 200 kHz. Each measurement point was repeated several times, and the average value was taken;
- Impedance measurement accuracy compared with SFB7. We applied impedance measurements with SFB7 and our device on an RRC network circuit at 5, 10, 50, 100, and 200 kHz. The RRC circuit consists of R1 in series with R2 ‖ C, with R1 = 22.1 Ω, R2 = 30.12 Ω, and C = 2.2 μF. The impedance measured with our device will be compared with the theoretical value and the measurement of SFB7;
- Settling time test to find out the best setting time which can be programmed in the embedded system;
- System speed performance. This test aims to derive the time needed by the system to measure the muscles’ EMG and EIM and send the data over UART or RF. This time is directly related to the maximum sampling frequency of the device. We used a logic analyzer (Az-Delivery, Germany) with 8 channels, sampling time 24 MHz, and the program Logic (Saleae, CA, USA) to observe the microcontroller’s correct communication with the modules, the interruption rate, and the values sent over UART or RF.

#### 2.4.2. Muscle Contraction Tests on a Healthy Subject

**Figure 9:**
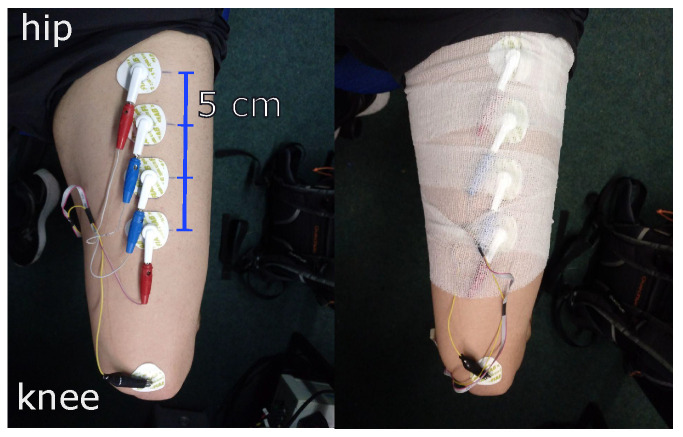
Placement of the electrodes on the upper leg. The ECG electrodes are placed with a distance of 5 cm from center to center (**left**). The electrodes are fixed with a bandage (**right**).

To demonstrate the device’s performance on a real subject during muscle contraction, one of the authors performed self-tests on himself within our lab. The target muscle for this measurement is the rectus femoris part of the quadriceps muscle. For this measurement, the test person attaches the electrodes to the target muscle. ECG electrodes are placed in a line along the leg with a distance of 5 cm measured from the center of the electrode. Next, the electrodes are fixed with a bandage to reduce the motion artifacts. Figure 9 shows how the electrodes are placed on the leg. Each procedure is repeated for different excitation signal frequencies at 2, 5, 10, 50, 100, and 200 kHz.

#### 2.4.3. Muscle Contraction on a Test Bench

**Figure 10:**
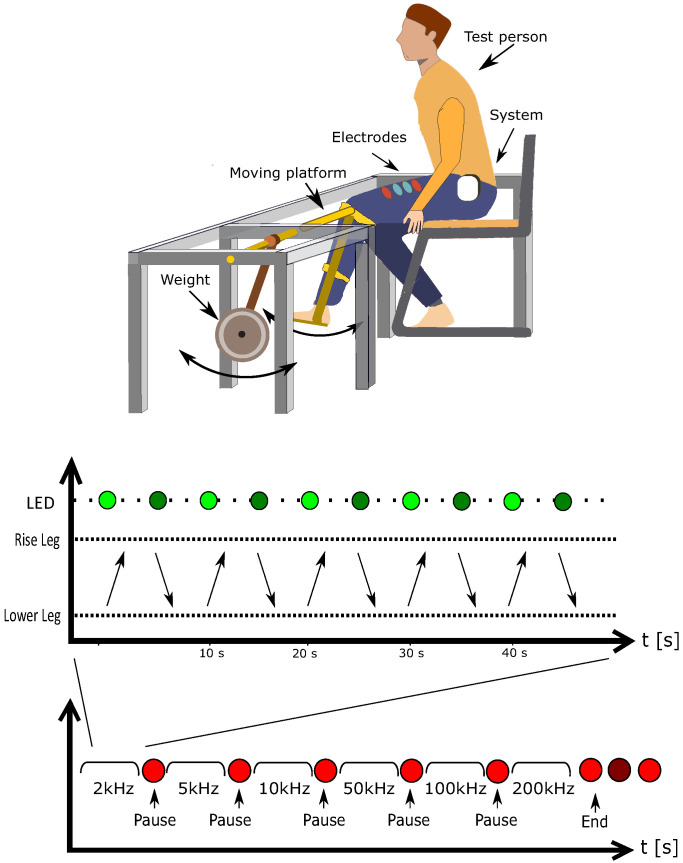
**Top**: setup of muscle contraction test on the test bench; **bottom**: protocol of muscle contraction test on the test bench.

The test person sits on a chair with the leg attached to a mechanical rotating test bench where the rotation axis is located close to the volunteer’s knee. The test bench allows the volunteer to contract the muscle while raising the leg from an angle of 90 degrees to 140 degrees. The structure was weighted with 7 kg loads. The test person raises and lowers the leg 5 times by himself or herself within approx. 50 s with a signal from a LED (red/green). After a short break, the test will be repeated for another measurement frequency. The setup and the protocol of this measurement are depicted in Figure 10.

#### 2.4.4. Sit-to-Stand Test with and without Weight

This test aims to investigate the EIM and EMG signal’s change while standing up and sitting down. For the subject, the test protocol is similar to the one shown in Figure 10, where “Raise leg” and “Lower leg” are replaced with “Stand up” and “Sit down”. At each frequency, the subject performed 5 times sit-to-stand. After finishing the first 2 normal sit-to-stands, another person helped to put a 15 kg backpack on his back. The subject performed this sit-to-stand 3 times with the weight.

#### 2.4.5. Up-and-Go Test

In this test, the test subject performed the following movements: standing up from a chair, standing still, then walking for about 20 s, then waiting and sitting back to the chair.

## 3. Results

### 3.1. System Performance: Filters Module

The HPF has a high CMRR regarding the choice of the components mismatch, a good SNR (simulated 150–160 dB), low noise (simulated total noise 40 nV), a cut-off frequency at 20 Hz, and is compatible with ADS1298. The LPF is a passive filter that is simple to realize; it has a cut-off frequency at 465 Hz, a good SNR (simulated 130–143 dB), and a low noise level (simulated total noise 280 nV).

### 3.2. EIM Module

**Figure 11:**
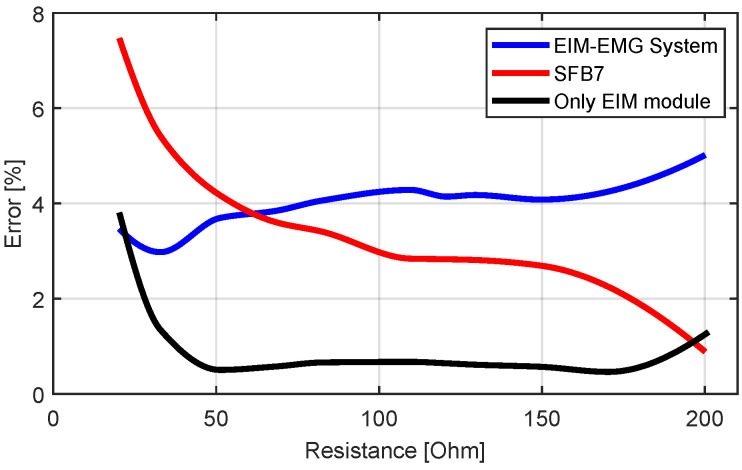
Percentage error of EIM module (black), EIM-EMG system (blue), and SFB7 (red) error respect to multimeter while measuring pure known resistances.

The accuracy test of the impedance measurement was performed both on the EIM module only (by disconnecting the EMG module) and on the combined EMG-EIM system. The calibration was done separately. The results are depicted in Figure 11.

The percentage error of the EIM module (in blue) lies under 0.8%, which outperforms the commercial SFB7. However, when the resistance approaches the range of the calibration resistances (20Ω and 200Ω), an increase in the error was observed. When combining EMG and EIM measurements, the overall error increased and lay around 4%, which is lower than the commercial SFB7 for small resistances <70 Ω and higher for resistances >70 Ω. The increase of the error could be explained by the noise produced by the path’s resistance since the EMG is attached between the EIM module and the electrodes.

**Figure 12:**
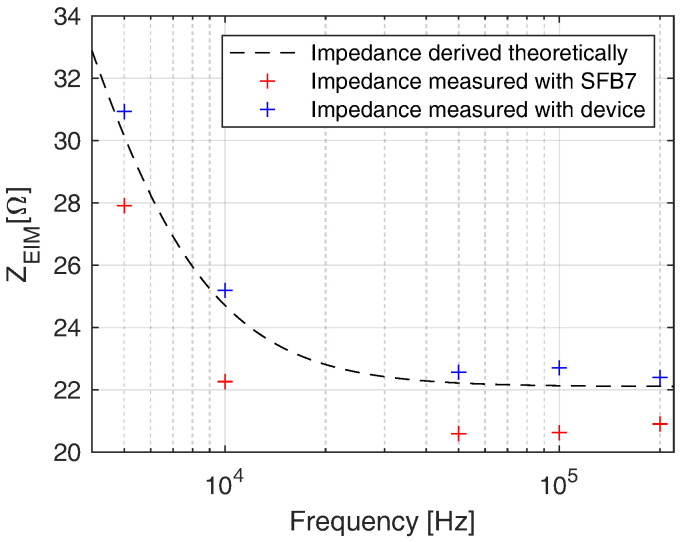
Impedance value of the RRC network over different frequencies. Values measured with our device (blue) are compared with values measured with SFB7 (red) and theoretical values (black).

Figure 12 depicts the measurement results of the RRC network at 5, 10, 50, 100, and 200 kHz. The values measured with our device and with the SFB7 are shown in blue and red, respectively. It can be seen that our system outperformed the commercial SFB7 at all applied frequencies. The mean errors between theoretical and measured values are 2% measured with our device and 7.38% measured with SFB7.

### 3.3. Frequency Response

**Figure 13:**
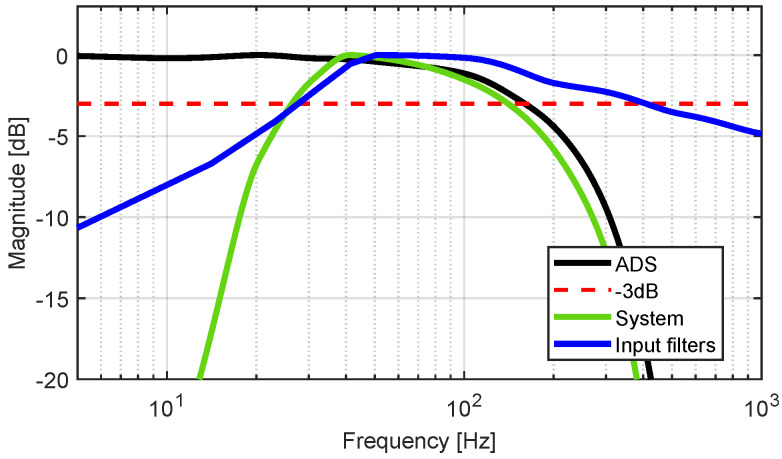
Frequency response of EMG module with filter.

The frequency response of the combination of the filters is depicted in blue in Figure 13, where the lower cut-off frequency $f_{c_{Filter,low}}$ is 20 Hz and the upper $f_{c_{Filter,up}}$ is 320 Hz. It can be observed that the upper cut-off frequency $f_{c_{ADS,up}}$ is 150 Hz. This cut-off frequency is limited by the sigma-delta ADC built in the ADS1298 and can be raised by increasing the sampling frequency of the ADC. The frequency response of the filters in combination with the EMG module is depicted in green. The upper cut-off frequency of the whole system is $f_{c_{Sys,high}}=150$ Hz, and the lower is $f_{c_{Sys,low}}=20$ Hz.

### 3.4. Settling Time

A settling time below 100 ms will cause an inappropriate error in the measured resistance value (above 20% compared to the value given by a standard multimeter). Settling times between 150 ms and 500 ms could be accepted as a trade-off between speed and accuracy. A value above 500 ms will not bring any further advantages to the accuracy of the measurement.

### 3.5. The Test-Bench Test for Muscle Contraction

**Figure 14:**
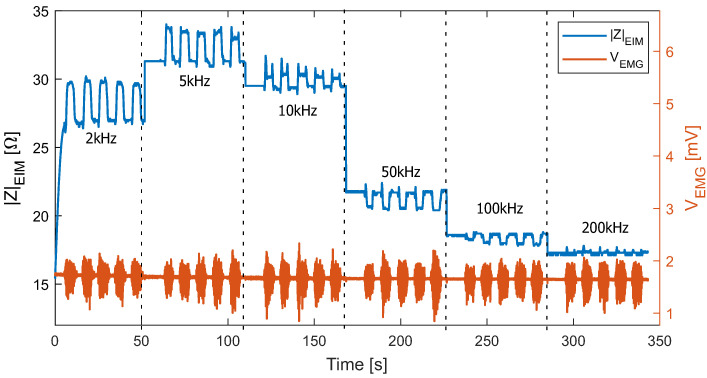
EIM of volunteer 1 during the contraction test with 7 kg. The EMG amplitude is higher during contraction.

The results of the test-bench test with 7 kg weight on a healthy volunteer are depicted in Figure 14. The change in EMG signal during muscle contraction and resting can be observed. The EIM measurement worked adequately at frequencies between 2 and 200 kHz.

Note that the bio-impedance value during resting is frequency-dependent. It reached its maximum value at 5 kHz, then decreased at increasing frequencies. During contraction, the amplitude change was maximum (about 3 Ω) at a lower frequency (2 kHz), decreased at increasing frequency. Between 10 and 50 kHz, it switched from positive to negative and became insignificant at higher frequencies.

**Figure 15:**
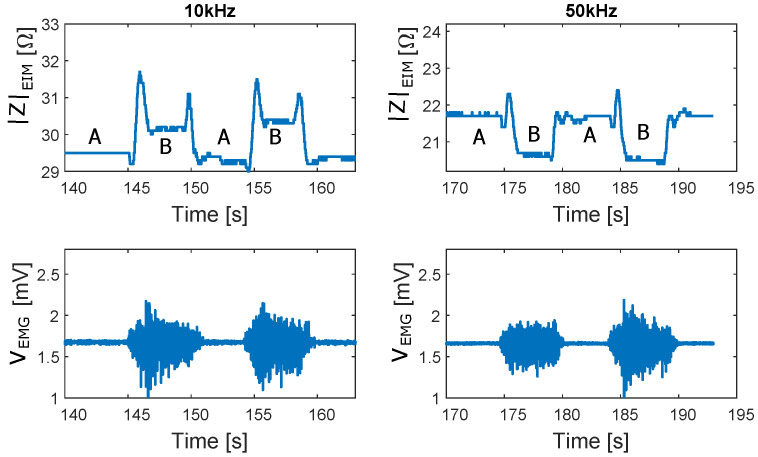
EIM and EMG signals measured from a test subject during movement with contraction. Measurement performed at 10 and 50 kHz. A: marks the period when the volunteer had their leg bent at 90 degrees. B: marks when the leg was elongated to 140 degrees.

For a better illustration, Figure 15 shows a segment of the EIM and EMG signals at 10 and 50 kHz. A marked the 90° position of the leg (resting) and B marked the 140° position of the leg (holding 7 kg weight at maximum deflection). While no differences can be observed within the EMG signals, a switch of the relation between the EIM values at A and B for 10 and 50 kHz can be observed. During the transition from A to B and from B to A, which corresponds to the movement phases, peaks can be observed in the EIM signals.

### 3.6. The Sit-to-Stand Test with and without Weight

**Figure 16:**
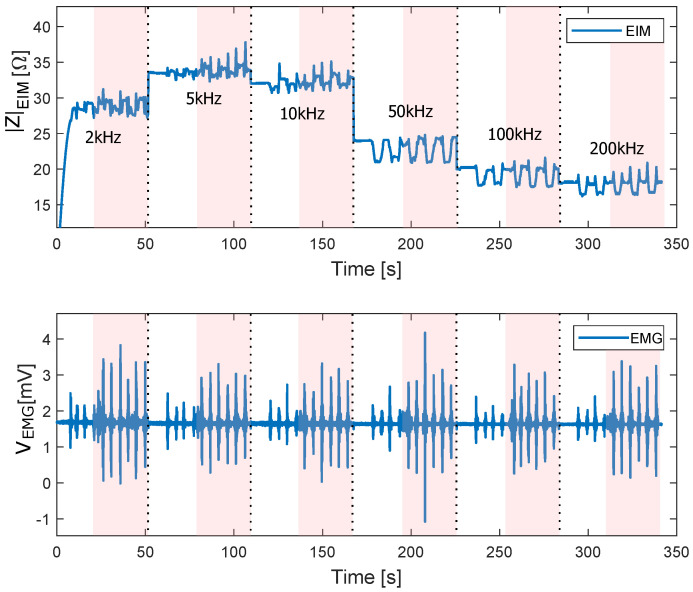
EIM and EMG at different frequencies during sit-to-stand with and without a weight of 15 kg attached to the back. The interval when the proband performed sit-to-stand cycle with 15 kg extra weight is marked in red. The sit-to-stand cycle without extra weight is marked in white.

The results of the sit-to-stand test with a load of 15 kg are depicted in Figure 16. The sit-to-stand cycles which the volunteer performed without extra weight are marked in white, while the sit-to-stand cycles with the extra weight are shadowed in red. In the case of the sit-to-stand test without weight, the plateau difference between contraction and without contraction (corresponding to the difference between A and B in Figure 15) becomes more evident at higher frequencies. It is easy to perceive that with extra weight, the peaks on the EIM signal become bigger. Moreover, the amplitude of the EMG signal also increases compared to the sit-to-stand cycles without extra weight. It can be observed how the EMG signal increases its amplitude at any frequency when the muscle exerts more force. The EIM signal, on the other hand, shows a higher difference between sitting and standing when the force increases.

### 3.7. The Up-and-Go Test

**Figure 17:**
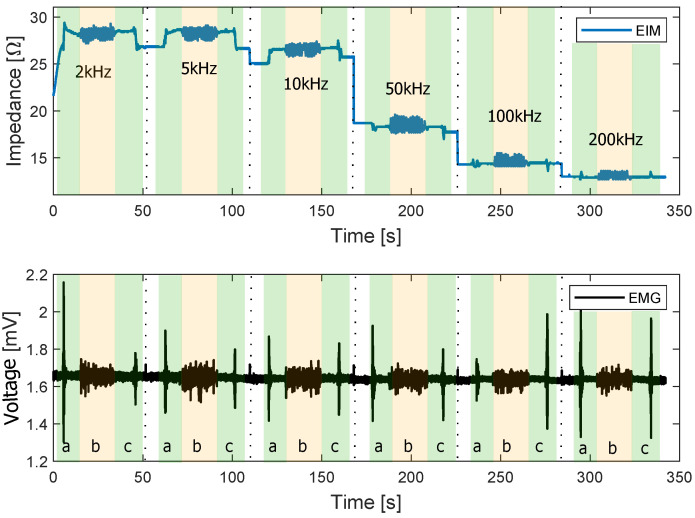
EIM and EMG signals over time at different frequencies during the sit-to-stand and walking phase. a: Sit-to-stand, b: walking, c: stand-to-sit.

The results of the walking test are presented in this section. Figure 17 depicts the EIM and EMG of the walking test performed at different frequencies. The transitions from sitting to standing (a) and from standing to sitting (c) are shadowed in green. Walking phases are shadowed in yellow (b).

It can be observed that the transitions from sit to stand and vice versa have a higher amplitude in the EMG signal. During the walking phase, the different peaks produced by each step of the volunteer are apparent. In addition, the EMG signal during this phase is small compared to the amplitude during sit-to-stand transitions. This indicates a lower activation of the target muscle during the walking phase than during sit-to-stand.

## 4. Discussion and Conclusions

The EIM module can measure bioimpedance between 20 Ω and 200 Ω with an error of less than 5% at 140 SPS and a settling time of less than 1000 ms. Compared with the commercial device SFB7, this device can provide similar accuracy and even better values for resistances lower than 50 Ω.

The EMG module captures the spectrum of the EMG signal between 20–150 Hz at 1 kSPS. The SNR of the EMG module is 67 dB. Moreover, the whole system reads and transfers the data of both modules every 1ms over RF (868 Mhz) with a baud rate of 500 kbps. These parameters partially fulfill the requirements, since the EMG module’s upper cut-off frequency could be improved by increasing the sampling rate.

The system’s accuracy and overall performance allowed us to perform measurements on a healthy volunteer during motion. The contraction investigation tests have shown that the EIM signal change at low frequencies is closely related to contraction. In contrast, the morphological change of the muscle has a more significant impact at high frequencies. The sit-to-stand test shows that the force exerted by the muscle influences the amplitude of both the EMG and EIM signals. Therefore, this system can be used as a new tool to investigate muscle condition and muscle force/torque.

The modular design allows combining the modules to detect several muscle condition features or perform simultaneous multi-frequency bioimpedance measurements. This new wearable hardware design was tested in depth. A significant effort has been made to optimize its overall performance to provide accurate and reliable measurements of the EMG and EIM signals. Therefore, this system can be used as a new tool to investigate muscle condition and muscle force/torque. Preliminary results show the system’s ability to measure muscle contraction with EIM and EMG during walking, sit-to-stand, and weight lifting. 

## Data Availability

Not applicable.
